# The impacts of *MACC1* gene polymorphisms on urothelial cell carcinoma susceptibility and clinicopathologic characteristics in Taiwan

**DOI:** 10.7150/jca.90130

**Published:** 2023-10-24

**Authors:** Li-Wen Chang, Sheng-Chun Hung, Ying-Erh Chou, Chuan-Shu Chen, Jian-Ri Li, Chia-Yen Lin, Shian-Shiang Wang, Shun-Fa Yang

**Affiliations:** 1 Institute of Medicine, Chung Shan Medical University, Taichung, Taiwan.; 2 Division of Urology, Department of Surgery, Taichung Veterans General Hospital, Taichung, Taiwan.; 3 Department of Post-Baccalaureate Medicine, College of Medicine, National Chung Hsing University, Taichung, Taiwan.; 4 School of Medicine, Chung Shan Medical University, Taichung, Taiwan.; 5 Department of Medicine and Nursing, Hungkuang University, Taichung, Taiwan.; 6 School of Medicine, National Yang Ming University, Taipei, Taiwan.; 7 Department of Applied Chemistry, National Chi Nan University, Nantou, Taiwan.; 8 Department of Medical Research, Chung Shan Medical University Hospital, Taichung, Taiwan.

**Keywords:** MACC1, single nucleotide polymorphism, urothelial cell carcinoma

## Abstract

Urothelial cell carcinoma (UCC) is a common malignancy of the urinary tract in Taiwan. Metastasis-Associated in Colon Cancer 1 (MACC1), a newly identified oncogene and regulator of the HGF/Met signaling pathway, has been shown to play a critical role in the development and progression of several types of cancer. Our study aims to investigate the impact of *MACC1* gene polymorphisms on the clinicopathological features of patients with UCC. In this study, we included a total of 719 patients with UCC and 719 healthy controls. The genotyping of five *MACC1* gene polymorphisms (rs1990172, rs975263, rs3095007, rs4721888, and rs3735615) was performed using real-time PCR with TaqMan assays. Our findings indicate that urothelial cancer patients with *MACC1* rs3095007 A allele had a decreased risk of >T2 stage [Odds ratio (OR)=0.619, 95% CI=0.394-0.971, *p*=0.036] and lymph node invasion (OR=0.448, 95% CI=0.201-0.998, *p*=0.044). Additionally, these individuals were associated with longer relapse-free survival (*p*=0.007) and overall survival (*p*=0.028). In conclusion, our findings demonstrate that urothelial cancer patients with *MACC1* (rs3095007) CA and AA genotypes have a lower risk of advanced T stage and lymph node metastasis. Additionally, these genotypes were associated with longer relapse-free survival and overall survival, highlighting the potential of these biomarkers as predictors of UCC prognosis.

## Introduction

Bladder cancer (BC) is the fourth most common cancer in males and sixth in both genders, accounting for 81,180 new diagnosis cases and 17,100 deaths in the USA in 2022 1. 95% of BC cases develop from the urothelium and are called urothelial cell carcinoma (UCC), which is categorized into non-muscle invasive bladder cancer and muscle invasive bladder cancer based on the depth of tumor invasion 2, 3. About 75% of UCC patients initially present with non-muscle invasive tumors, which are confined to the mucosa as stage Ta or carcinoma in situ (CIS) or confined to the submucosa as stage T1. The remaining patients present with muscle invasive tumors, defined as stage T2-4 disease, and have a higher risk of cancer-specific death 4, 5.

Tobacco smoking is the most significant risk factor for UCC and accounts for approximately half of all UCC cases 6. Environmental or occupational exposure is the second most important risk factor and accounts for 10% of cases 7. Genetic predisposition may also increase the susceptibility to UCC 8-12. For example, TP53 mutation is found in almost 50% of bladder cancer patients and is associated with disease progression and drug selection 13. Currently, data from genome-wide association studies have also identified three single nucleotide polymorphisms (SNPs) associated with aggressive UCC and a higher risk of progression 14. However, there is still insufficient evidence to endorse genetic screening for UCCs.

Metastasis associated in colon cancer 1 (MACC1) gene was first identified in 2009 as a key regulator of hepatocyte growth factor-mesenchymal-epithelial transition factor (HGF-MET) signaling and the expression of MACC1 in tumor specimens is an independent prognostic factor for colon cancer 15. The HGF-MET signal pathway activates the ERK/MAPK pathway and PI3K/Akt/mTOR pathway for cell proliferation and survival, leading to cancer proliferation, angiogenesis, tumor invasion, and metastasis 16, 17. Upon binding of HGF to its receptor MET, MACC1 undergoes translocation from the cytoplasm to the nucleus 15. MACC1 then binds to the promoter of the MET gene to increase the transcription and generate more MET protein as a receptor for HGF. Thus, the HGF-MET-MACC1 axis results in abnormal cell proliferation and an increased ability of cancer migration, invasion, and metastasis 18-20. MACC1 overexpression could contribute to colorectal cancer progression and metastasis 18. Similar results have been found in lung, gastric, liver, and ovarian cancer 21-24. MACC1 expression is highly associated with lymphatic metastasis in oral cancer, and downregulation of MACC1 inhibits the migration, and proliferation of tumors 25. MACC1 expression is also an independent predictor of more advanced tumor stage, grade of differentiation, and lymph node metastasis in infiltrating urothelial cell carcinoma of the bladder 26.

Variations in a solitary nucleotide base of the DNA sequence, known as single nucleotide polymorphisms (SNPs), are present in the population at a frequency of at least 1% 27, 28. These genetic variations can result in amino acid substitutions that affect protein function and contribute to disease development 29-31. For instance, the G allele of rs1990172 at *MACC1* has been linked to significantly decreased overall survival in colorectal cancer, while heterozygous carriers of SNPs rs1990172 and rs975263 showed a significantly higher risk of disease relapse in hepatocellular carcinoma recurrence in liver transplant patients 32, 33. Similarly, carriers of the G allele of SNP rs1990172 had an increased risk of progression and death in HER2-positive breast cancer patients, whereas the C allele of SNP rs3735615 conferred significant protective impact on overall survival 34.

Despite the known association of *MACC1* SNPs with various cancers, their role in patients with UCC has not been established. In this study, we examined five MACC1 SNPs (rs1990172 [intron], rs975263 [exon 5], rs3095007 [intron], rs4721888 [exon 4], rs3735615 [exon 7]) to investigate their predictive role in UCC patients.

## Material and methods

### Subjects and Specimen Collection

Between 2011 and 2022, Taichung Veteran General Hospital in Taichung, Taiwan, conducted a case-control study that enrolled a total of 719 patients with UCC, consisting of 441 men and 278 women. To serve as a control group, 719 individuals of the same ethnic background but without a history of cancer of any sites were also enrolled. The patients were diagnosed with UCC based on the TNM staging system of the American Joint Committee on Cancer (AJCC) Staging Manual (7th ed.), which was confirmed by pathologists 35. Tumor histopathologic grading was based on the 2004 WHO grading system, which classified tumors as either high grade or low-grade papillary tumors 36. Lymph node and metastasis assessments were carried out using regular computer tomography (CT) scans. Personal information and patient characteristics were obtained through interviewer-administered questionnaires, which included questions about demographics and cigarette smoking status. The Institutional Review Board (IRB) of Taichung Veterans General Hospital approved the study, and all participants provided informed written consent (IRB No. CE19106A). Ethylenediaminetetraacetic acid (EDTA)-containing tubes were used to collect whole-blood specimens from both control and patient groups, which were immediately centrifuged and stored at -80°C.

### *MACC1* Polymorphism Selection

To investigate the relationship between *MACC1* and UCC development, five well-characterized common polymorphisms were selected for this study, based on their association with cancer development and staging 32, 34, 37, 38. The SNP rs1990172 is located at an intronic region of the *MACC1* gene and has been associated with overall survival in colorectal cancer patients 32. The SNP rs3095007, located at an intron of the *MACC1* gene, is one of the most common variants representing the majority of the *MACC1* locus 39. Meanwhile, the SNPs rs975263, rs4721888, and rs3735615, which are located at exons 5, 4, and 7, respectively, were associated with breast cancer susceptibility and clinical outcomes 34, 37.

### Genomic DNA extraction

DNA was extracted from whole blood samples using QIAamp DNA blood mini kits, and stored at -20°C in TE buffer until Real-time quantitative PCR analysis was performed 40. The allelic discrimination of the *MACC1* rs1990172, rs975263, rs3095007, rs4721888, and rs3735615 polymorphisms was assessed using the ABI StepOne TM Real-Time polymerase chain reaction (PCR) System (Applied Biosystems, Foster City, CA), and the data were analyzed using SDS v3.0 software (Applied Biosystems, Foster City, CA).

### Statistical Analysis

The differences in demographic characteristics between the control and UCC group were compared using the Chi-squared test. Multiple logistic regression models were used to assess the odds ratios (ORs) with 95% confidence intervals (CIs) for the association between genotype frequencies and clinicopathologic characteristics, controlled with other covariates. Disease susceptibility was adjusted by multiple logistic regression models after controlling for age, sex and tobacco consumption. The Kaplan-Meier survival curve and multiple Cox proportional hazards model were used to evaluate disease-specific mortality and all-cause of death. A *p* value of less than 0.05 was considered statistically significant. The Statistical Analytic System (SAS Institute, Cary, NC, USA) software for Windows was used for all data analysis.

## Results

### Characteristics of Study Participants

The study cohort comprised 719 patients with UCC and 719 matched healthy controls. Table 1 showed the mean age of patient with UCC was 60.65 ± 7.07 and 68.86 ± 11.53 in control (*p*<0.001). Male predominance was observed in UCC patients (n=441, 61.3%), with no significant difference in gender distribution between the two groups (*p*=1.000). At diagnosis, 49.0% (n=352) of UCC patients were diagnosed with stage 1 disease, while 51.0% (n=367) had stage 2-4 disease. Pathological evidence of lymph node metastasis was found in 12.2% (n=88) of patients, while 3.6% (n=26) had confirmed metastatic disease. Histopathological analysis revealed that 89.3% (n=642) of UCC patients had high-grade tumors, and 10.7% (n=77) had low-grade tumors.

### Association of *MACC1* Gene Polymorphisms with UCC

Table 2 illustrates the genotype distributions of the *MACC1* gene, revealing that homozygous CC at rs1990172, homozygous AA at rs975263, homozygous GG at rs3735615, homozygous GG at 4721888, and homozygous CC at rs3095007 exhibit the highest distribution frequencies. However, there is no significant difference observed between *MACC1* gene polymorphisms and susceptibility to UCC (Table 2).

### Correlation between *MACC1* SNPs and Clinical Status of UCC

**Figure 1:**
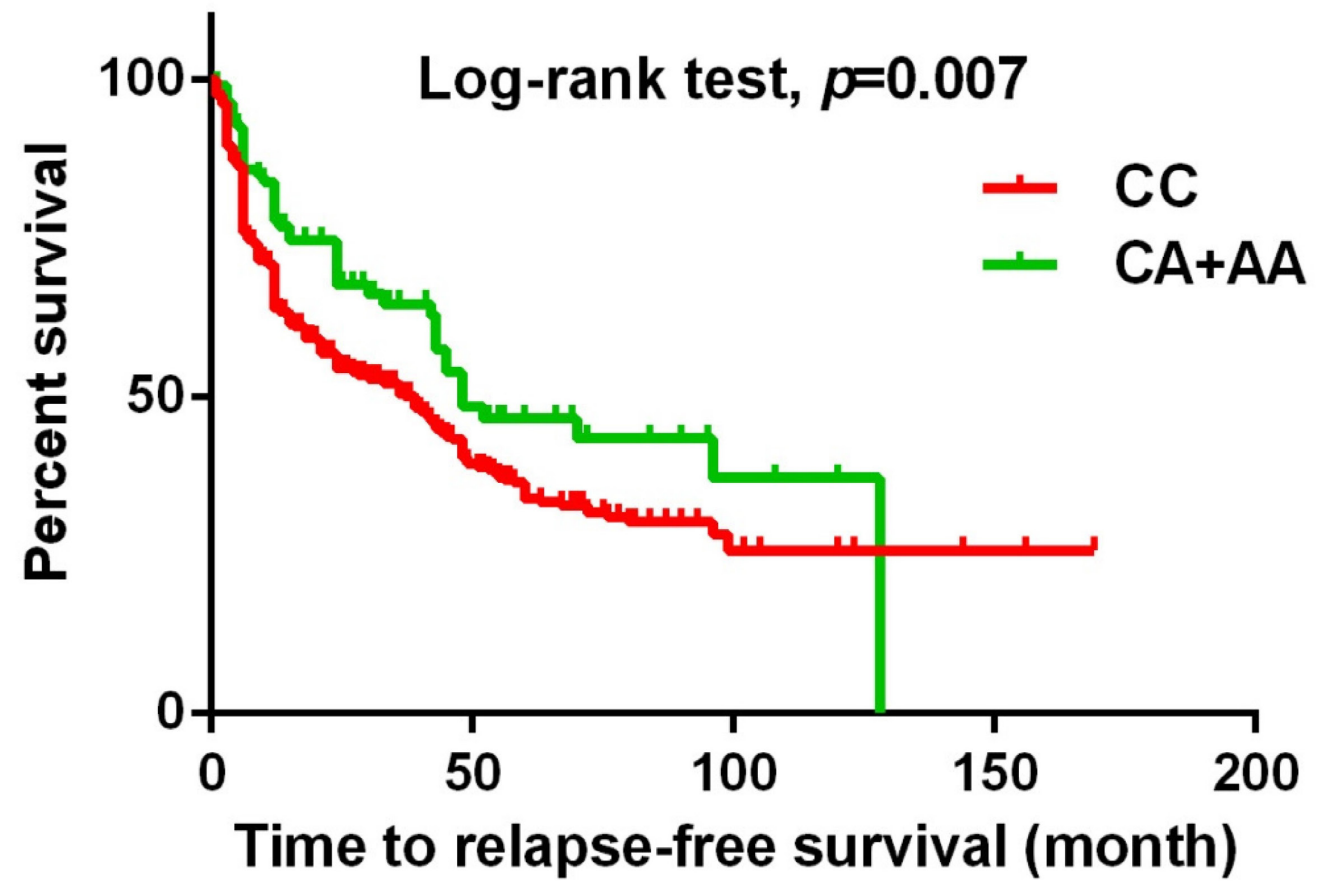
** The Kaplan-Meier survival curve was used to analyze the relapse-free survival for 719 patients with UCC.** Patients with CA+AA allele at rs3095007 has a longer relapse free survival compared to patients with CC allele (*p*=0.007).

**Figure 2:**
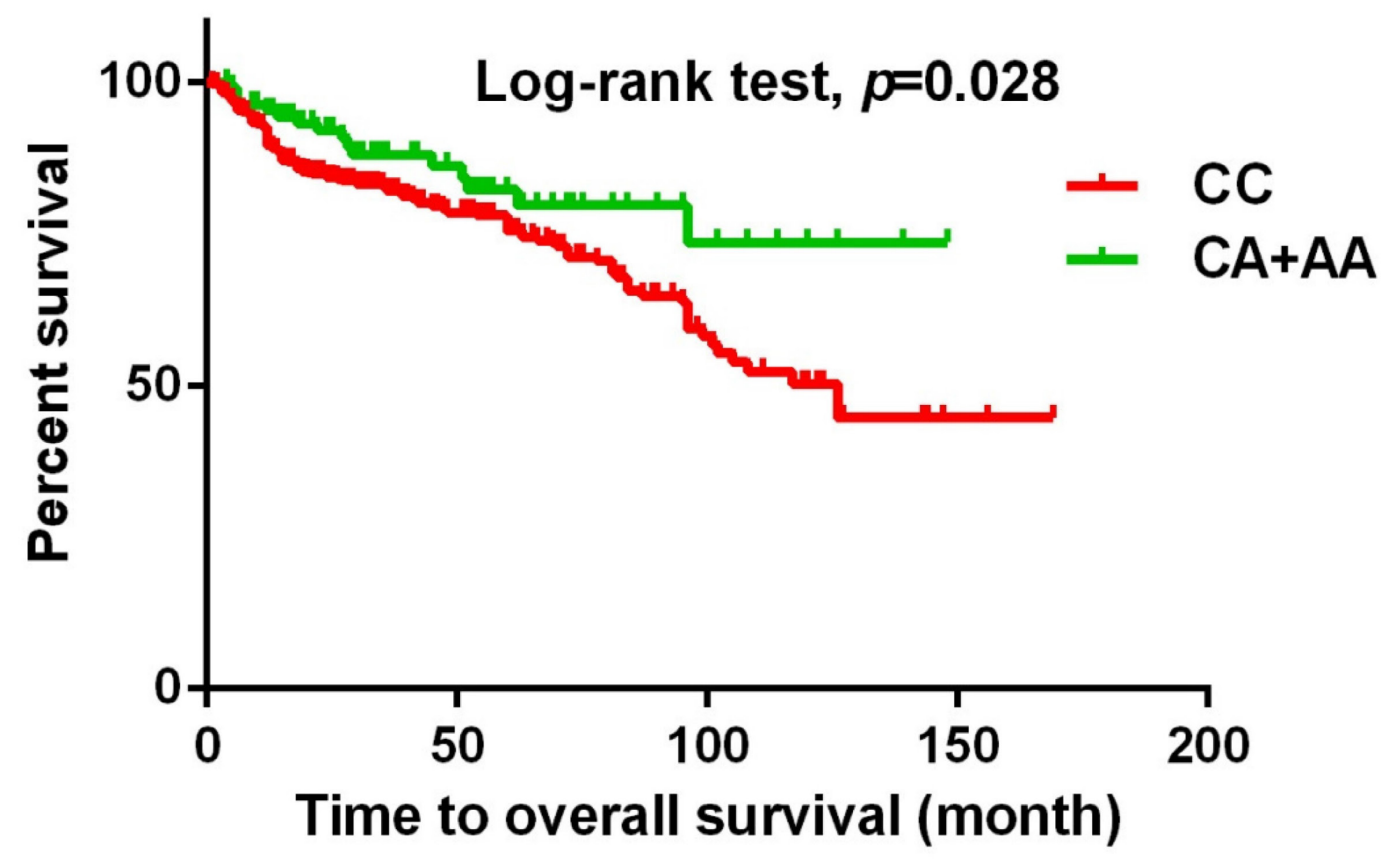
** Kaplan-Meier survival curve was used to analyze the overall survival for 719 patients with UCC.** Patients with CA+AA allele at rs3095007 has a longer overall survival compared to patients with CC allele (*p*=0.028).

The present study investigated the association between *MACC1* SNPs and clinicopathologic characteristics of UCC patients. Table 3 displays patients with at least one A allele at rs3095007 exhibit a lower proportion of advanced lesions (>T2 status) (OR = 0.619, 95% CI = 0.394-0.971, *p* = 0.036) and less lymph node metastasis disease (OR = 0.448, 95% CI = 0.201-0.998, *p* = 0.044). Furthermore, Figure 1 demonstrates that the presence of at least one A allele at *MACC1* rs3095007 is linked to longer relapse-free survival (*p* = 0.007), while Figure 2 reveals that this allele is associated with longer overall survival (*p* = 0.028).

## Discussion

The current study represents the initial investigation into the correlation between *MACC1* SNPs and clinicopathologic characteristics as well as prognosis of UCC in the Chinese population. Our findings suggest that UCC patients who possess at least one A allele at rs3095007 exhibit less advanced tumor and lymph node metastasis, as well as a prolonged relapse-free survival and overall survival. To our knowledge, this is the first report to document these associations in this patient population.

Overexpression of MACC1 has been demonstrated to upregulate the HGF-MET signaling pathway, which in turn promotes tumor proliferation, invasion, and metastasis in colorectal cancer 15. Furthermore, elevated MACC1 mRNA expression in lung adenocarcinoma specimens is associated with early recurrence following surgery 21. In breast cancer specimens, MACC1 protein expression is linked to advanced pathological features, reduced relapse-free survival, and overall survival rates 41. In UCC tissue, MACC1 is more frequently expressed than in normal bladder mucosa tissue, and its expression is positively associated with tumor stages, grades of differentiation, lymph node metastasis, stages, and overall unfavorable survival rates 26. RNA interference-mediated knockdown of MACC1 gene expression in T24 cells (human bladder urothelial cell carcinoma cells) resulted in decreased proliferation, expression of apoptosis proteins, and downregulated MET protein levels, thus reducing the invasion abilities of T24 cells 42. These findings indicate that MACC1 may serve as a promising prognostic indicator or a potential therapeutic target for gene therapy in human UCC.

The promoter activity and gene expression of MACC1 can be influenced by genetic polymorphisms, which may potentially affect tumor growth, invasion, or metastasis 43-46. Lang et al. conducted the first study to investigate the association between *MACC1* SNPs and the survival of colorectal cancer patients. They genotyped 6 tag SNPs located in the intronic region, which represent the majority of common variants at the *MACC1* locus. Their findings showed that the rs1990172 SNP was considerably associated with decreased overall survival 32. Schmid et al. further sequenced the coding exons of *MACC1*, including three SNPs (rs4721888, rs975263, rs3735615). They found that younger patients with colon cancer with stage I or II and a CT genotype at rs975263 had a shorter metastasis-free survival. However, in cell culture experiments, *MACC1* SNPs have no impaction on MACC1-induced cell migration and proliferation 38. Consistent with Lang et al., Horvat et al. reported that the TT genotype of SNP rs1990172 in the *MACC1* gene was associated with worse disease-free survival in resectable colorectal cancer patients 47.

Similar results have been reported in other types of cancer. For instance, Zheng et al. found that patients with hepatocellular carcinoma who were heterozygous for the rs1990172 SNP in the intronic region or for the rs975263 SNP in the exon region had a significantly higher risk of relapse after transplantation 33. Lin et al. also reported that patients with hepatocellular carcinoma who carried the CA or AA variant at rs1990172 had a lower risk of developing larger tumors, more advanced clinical stages, and vascular invasion 48. Muendlein et al. found that carriers of the rare G allele at rs1990172 and the rare T allele at rs975263 had an increased risk of disease progression and death in patients with HER2-positive breast cancer 34. In another cohort study of breast cancer in Han Chinese women, Dai et al. did not find any associations between rs1990172 and breast cancer risk, but they did observe that rs975263 and rs472188 in the exon region were associated with susceptibility to breast cancer in Chinese women 37. Moreover, Hu et al. revealed that rs975263 may have the potential to be a metastasis marker in oral cancer patient 49. Similarly, Sun et al. found that Taiwanese women with cervical cancer who had the GG genotype at rs975263 tended to have a higher risk of vaginal invasion than those with AA/AG variants 50.

In our study of UCC patients, we examined two intronic SNPs (rs1990172 and rs3095007) and three exonic SNPs (rs975263, rs4721888 and rs3735615). While rs1990172 is located within an intronic region, previous studies have shown its significant impact on disease prognosis in colorectal cancer, breast cancer, and hepatocellular carcinoma 32-34. This may be attributed to the fact that introns can affect various aspects of gene expression, including transcription rate, nuclear export, transcript stability, and mRNA translation efficiency 51. However, we did not observe significant impact of rs1990172 on disease prognosis in our population. Instead, we found that at least one A allele at rs3095007, also located in an intronic region, was associated with a lower risk of advanced tumor stage and lymph node metastasis, as well as longer progression-free survival and overall survival. This is the first report of a positive association between rs3095007 and disease prognosis, although the underlying mechanism is yet to be investigated. Notably, we did not observe any clinical significance of the three exons in UCC patients.

There are several limitations in our study that need to be acknowledged. Firstly, a larger cohort of case-control analysis is necessary to confirm our findings. Although we observed a trend of higher UCC susceptibility in the three exonic SNPs (GG for rs975263, CC for rs3735615, and CC for rs4721888), statistical significance was not reached. Therefore, more cases may be needed to explore the role of these three coding exons in UCC susceptibility. Secondly, the precise mechanism by which these SNPs affect the function of the *MACC1* gene requires further investigation. Thirdly, the treatment modality of the patients was not taken into consideration in our study, which may have an impact on the prognosis and outcome of the disease.

In conclusion, this study is the first to report the relationship between *MACC1* polymorphism and the clinicopathologic features of UCC. Our findings demonstrate that urothelial cancer patients with *MACC1* (rs3095007) CA and AA genotypes have a lower risk of advanced T stage and lymph node metastasis. Additionally, these genotypes were associated with longer relapse-free survival and overall survival, highlighting the potential of these biomarkers as predictors of UCC prognosis.

## Figures and Tables

**Table 1:** The distributions of demographical characteristics in 719 controls and 719 patients with UCC.

Variable	Controls (N=719)n (%)	Patients (N=719)n (%)	*p* value
Age (yrs)			
Mean ± S.D.	60.65 ± 7.07	68.86 ± 11.53	<0.001
Gender			
Female	278 (38.7%)	278 (38.7%)	1.000
Male	441 (61.3%)	441 (61.3%)	
Stage			
Stage 1		352 (49.0%)	
Stage 2-4		367 (51.0%)	
Tumor T status			
≤ T2		457 (63.6%)	
> T2		262 (36.4%)	
Lymph node status			
N0		631 (87.8%)	
N1+N2		88 (12.2%)	
Metastasis			
M0		693 (96.4%)	
M1		26 (3.6%)	
Histopathologic grading			
Low grade		77 (10.7%)	
High grade		642 (89.3%)	

Student's t test or Chi-squared test was used between controls and patients with UCC.

**Table 2:** Genotype distributions of *MACC1* gene polymorphisms in 719 controls and 719 patients with UCC.

Variable	Controls (N=719) n (%)	Patients (N=719) n (%)	AOR (95% CI)	*p* value
**rs1990172**				
CC	539 (75.0%)	543 (75.5%)	1.000 (reference)	
CA	169 (23.5%)	158 (22.0%)	0.969 (0.740-1.271)	0.822
AA	11 (1.5%)	18 (2.5%)	1.747 (0.747-4.085)	0.198
CA+AA	180 (25.0%)	176 (24.5%)	1.014 (0.780-1.318)	0.917
**rs975263**				
AA	502 (69.8%)	487 (67.7%)	1.000 (reference)	
AG	198 (27.5%)	204 (28.4%)	1.058 (0.821-1.363)	0.663
GG	19 (2.7%)	28 (3.9%)	1.848 (0.956-3.573)	0.068
AG+GG	217 (30.2%)	232 (32.3%)	1.119 (0.877-1.428)	0.364
**rs3735615**				
GG	521 (72.5%)	511 (71.1%)	1.000 (reference)	
GC	185 (25.7%)	188 (26.1%)	1.084 (0.835-1.405)	0.545
CC	13 (1.8%)	20 (2.8%)	2.027 (0.919-4.471)	0.080
GC+CC	198 (27.5%)	208 (28.9%)	1.138 (0.884-1.464)	0.317
**rs4721888**				
GG	379 (52.7%)	376 (52.3%)	1.000 (reference)	
GC	290 (40.3%)	275 (38.2%)	0.935 (0.737-1.188)	0.584
CC	50 (7.0%)	68 (9.5%)	1.432 (0.985-2.659)	0.075
GC+CC	340 (47.3%)	343 (47.7%)	1.041 (0.830-1.305)	0.730
**rs3095007**				
CC	595 (82.8%)	610 (84.8%)	1.000 (reference)	
CA	118 (16.4%)	103 (14.4%)	0.933 (0.682-1.278)	0.666
AA	6 (0.8%)	6 (0.8%)	0.952 (0.282-3.217)	0.937
CA+AA	124 (17.2%)	109 (15.2%)	0.934 (0.687-1.270)	0.664

The adjusted odds ratio (AOR) with their 95% confidence intervals were estimated by multiple logistic regression models after controlling for age, gender and tobacco consumption.

**Table 3:** Distribution frequency of the clinical status and *MACC1* rs3095007 genotype frequencies in 719 UCC patients.

	*MACC1* (rs3095007)			
Variable	CC (%) (n=610)	CA + AA (%) (n=109)	OR (95% CI)	*p* value
**Stage**				
Stage 1	294 (48.2%)	58 (53.2%)	1.000 (reference)	
Stage 2-4	316 (51.8%)	51 (46.8%)	0.818 (0.544-1.231)	0.335
**Tumor T status**				
≤ T2	378 (62.0%)	79 (72.5%)	1.000 (reference)	
> T2	232 (38.0%)	30 (27.5%)	**0.619 (0.394-0.971)**	**0.036**
**Lymph node status**				
N0	529 (86.7%)	102 (93.6%)	1.000 (reference)	
N1+N2	81 (13.3%)	7 (6.4%)	**0.448 (0.201-0.998)**	**0.044**
**Metastasis**				
M0	588 (96.4%)	105 (96.3%)	1.000 (reference)	
M1	22 (3.6%)	4 (3.7%)	1.018 (0.344-3.014)	0.974
**Histopathologic grading**				
Low grade	62 (10.2%)	15 (13.8%)	1.000 (reference)	
High grade	548 (89.8%)	94 (86.2%)	0.709 (0.387-1.298)	0.263

Bold font indicates statistical significance (*p* < 0.05). The odds ratio (OR) with their 95% confidence intervals were estimated by logistic regression models.

## Abbreviations

BC: Bladder cancer
MACC1: Metastasis-Associated in Colon Cancer 1
SNP: single-nucleotide polymorphism
UCC: Urothelial cell carcinoma
